# Pain Management Associated with Posttraumatic Unilateral Temporomandibular Joint Anterior Disc Displacement: A Case Report and Literature Review

**DOI:** 10.1155/2018/8206381

**Published:** 2018-04-17

**Authors:** Arturo Garrocho-Rangel, Andrea Gómez-González, Adriana Torre-Delgadillo, Socorro Ruiz-Rodríguez, Amaury Pozos-Guillén

**Affiliations:** Pediatric Dentistry Postgraduate Program, Faculty of Dentistry, San Luis Potosi University, San Luis Potosi, SLP, Mexico

## Abstract

The aim of the present article is to review the etiological risk factors and the general and oral management of anterior disc displacement with reduction caused by a chin trauma, and to describe the diagnostic process and the treatment provided to an affected 7-year-old girl. The patient also experienced frequent and severe cephaleas, which may be related to cervical vertebrae deviation. The patient was successfully treated with an intraoral occlusal splint and analgesics. Pediatric dentists must always be aware of the early signs and symptoms of temporomandibular joint disorders in their patients, especially in cases of orofacial trauma history, with the aim of providing an opportune resolution and preventing its progression later in life. Occlusal splints are strongly recommended for the treatment of anterior disc displacement with reduction in children and adolescents.

## 1. Introduction

Temporomandibular disorders (TMDs) include a variety of pathological, single or combined, signs or symptoms of the temporomandibular joint and periarticular structures (masticatory muscles, ligaments, bone, and facial skin) [1]. These disorders affect not only adult patients; children also exhibit high incidence/prevalence of TMD, which have been associated with orofacial pain or discomfort, growth abnormalities, and mandibular dysfunction [2–4]. Some cases can course asymptomatic. Misdiagnoses are also frequent, confounding the clinical scenario as a chronic headache or otalgia. In other clinical situations, joint noises such as clicking, popping, snapping, or soft/hard-tissue crepitus are often perceived as normal by the general population and even by general dentists or physicians [2].

The TMD etiology in children and adolescents has been investigated by diverse authors without reaching any definitive consensus [5–7]. However, it is generally accepted that the disorder is of multifactorial origin, in which several genetic and/or environmental factors (e.g., systemic anomalies, parafunctional habits, psychological distress, anatomical factors, malocclusions, and local infection or trauma) can be related [1, 8, 9]. These etiological factors involve abnormal biomechanical forces applied to the mandibular condyle, altering the shape and function of the articular structures [10, 11]. The principal malocclusions related to TMD in children and adolescents are skeletal anterior open bite, steep articular eminence of the temporal bone, overjet > 6-7 mm, Class III malocclusion, and posterior crossbite [12].

Prevalence of TMD in children and adolescents has been estimated to be in the wide range of 16 to 68%, according to epidemiological studies from different countries; this variability is due to each study's methodology and the clinical features examined for determining the presence of the disorder [4, 8, 9]. The American Academy of Orofacial Pain [1, 13] has classified the TMD in children and adolescents into two broad categories: TMJ disorders—joint pain, joint disorders, and joint diseases—and masticatory muscle disorders. Joint disorders include the disc-condyle complex position disorders, also called as internal derangements (IDs). ID refers to an abnormal positional relationship of the articular disc in relation to the mandibular condyle and the articular eminence in the glenoid fossa, in the temporal bone [5, 10, 11, 14]. Although there are eight different abnormal disc positions, the anterior and anterior-lateral displacements are the most common. In turn, anterior disc displacements are classified into two main subgroups: displacement with reduction (ADDR) and displacement without reduction [14]; ADDR is the most frequent TMJ disorder found in children, with a prevalence of approximately 6% between 8 and 15 years old, with greater incidence in women [11].

The aim of the present report is to describe the clinical management provided to a 7-year-old girl with a TMD due to a chin trauma, specifically an anteroposterior joint disc displacement with reduction of interarticular space width, and related severe cephalea episodes, and the course of the abnormal condition over a six-month follow-up period.

## 2. Case Report

### 2.1. History of Complaints

A 7-year-11-month girl presented with her parents to the Pediatric Dentistry Clinic referred by a local general dentist, complaining of unilateral pain in the left TMJ area. The parents noticed an orofacial trauma episode: the child suffered a chin injury due to a drop from the bicycle at 4 years old, causing a significant skin abrasion/bruise and cervical sprain. The patient was not examined by a dentist, but we can speculate that there were no condylar fractures. An emergency physician indicated to use a semirigid neck collar for two weeks. Thereafter, the child experienced chronic pain in the left side of the face, around the temporomandibular zone, accompanied with recurrent moderate-to-severe cephalea episodes and mild symptoms of anxiety. A severe pinch-type pain during mastication of hard foods was also reported. She received pharmacological treatment, according to the age, weight, and size of the child, consisting in risperidone and imipramine for two weeks. However, the cephalea and intraoral pain had intensified during the last weeks; so, the parents decided to seek dental treatment. Paranormal habits were informed, particularly diurnal/nocturnal bruxism and onychophagy. Also, a previous event of transient jaw locking while eating was reported.

### 2.2. Extraoral and Intraoral Examinations

The clinical findings included normal stature and weight for her age. Her face was ovoid and symmetric with normal forehead and normal-set ears, and convex profile due to a retruded chin. Intraorally, the patient exhibited well-shaped arches in mixed dentition stage, multiple nondeep carious lesions, and Class I malocclusion with mild anterior crowding. No dental wear was observed.

**Figure 1:**
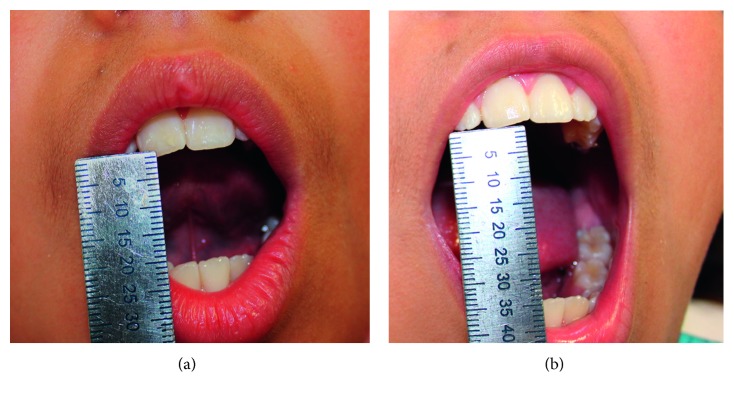
Oral aperture. Pretreatment (a). Posttreatment, taken after ten months of follow-up (b).

The maximum interincisal opening was only 20 mm (Figure 1(a)) (normal = 41–50 mm) [9, 15]. An evaluation of mandible function was carried out, following recommendations [16]; an evident mandibular left displacement was detected during the mouth opening/closure movements. A clicking sound was detected in the same joint, during mandibular opening, on stethoscope auscultation. Articular pain and masticatory muscle tenderness were assessed by bilateral extra and intraoral palpation, according to the Rocabado pain map criteria [17]; left temporomandibular joint manifested a moderate-to-severe posterior inferior synovial pain, radiating to the neck, indicating hurt by compression of the backward-positioned condyle.

### 2.3. Imaging

**Figure 2:**
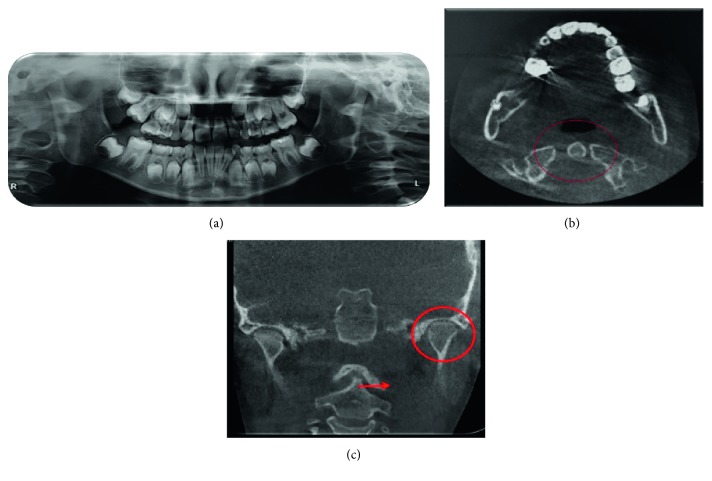
(a) Panoramic view. (b) CT of the cranial coronal view. In the circle, it can be observed the coronoid apophysis markedly deviated to the left, maybe caused by the reported craniofacial trauma. (c) CT anteroposterior view of the skull. The cycle shows the reduced interarticular space of the left temporomandibular joint, compared with the right side. The arrow represents the evident left deviation of the coronoid apophysis.

Radiographs and a computed tomography (CT) were taken. These images showed a reduction of the left articular condyle-glenoid fossae space width, with no evidence of condyle neck fracture, and a right deviation of first (odontoid process), second, and third cervical vertebrae, regarding the skull base, caused probably by the chin injury (Figure 2). This vertebrae deviation was compressing the spinal cord, giving origin to the moderate-to-severe cephalea episodes (cervicogenic cephalea), and perpetuating the temporomandibular dysfunction.

### 2.4. Treatment Approach

After performing a careful clinical diagnosis of ADDR, the dental treatment consisted in the placement of an intraoral removable soft occlusal splint covering the maxilla with an inserted midline bidirectional expansion screw to promote the transverse development. The patient's parents were fully explained about this therapeutic approach, and they agreed to sign a special informed consent form, which included the permission for publishing the clinical case.

**Figure 3:**
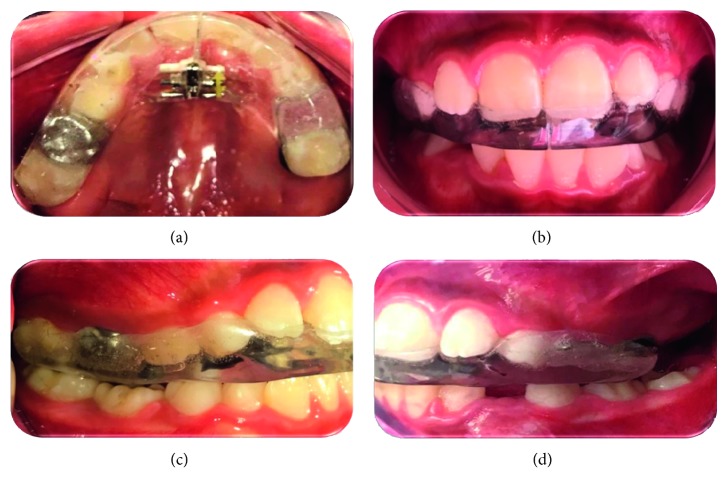
Different views of the intraoral appliance.

The appliance was fabricated with soft polyester and fitted over the upper arch teeth's occlusal and incisal surfaces, with a 2-3 mm thickness, in order to create a precise occlusal contact with the opposite teeth (Figure 3). The fabrication process was as follows: upper and lower stone models were mounted on an AD2 articulator in centric relation. This relation was previously taken in the patient, through adhesive anterior and posterior softened wax discs—for both upper first permanent molars and incisor registry, respectively—placed on a special metallic U-shaped bite plate. The plate was attached to a face bow, which was properly positioned over the patient's forehead. The patient was briefly trained to slowly bite, in centric relation, into the wax discs about 1 mm deep. After seating and fitting the intraoral appliance, simultaneous and symmetric contact points were obtained with maximum intercuspation and flat occlusal plane, according to the recommendations emitted by Restrepo et al. [18]. The patient was instructed to use the splint at least 12 hours a day, especially overnight, and to activate the expansion screw once, two days a week, in order to compensate the natural maxilla transverse growth.

The cervicogenic cephalea was managed with analgesics (Ibuprofen, 10 mg/kg/day orally, every 8 hours), and she was recommended to maintain a good posture when sitting and standing and avoid sleeping on her front, in order to reduce the strain on the neck muscles and ligaments. The parents were indicated to change their girl's pillow—for a softer one—and to place a rolled towel under her neck while sleeping. After two weeks, analgesic therapy was retired. The parents were instructed to apply a hot water bag wrapped in a towel directly to the affected head area, for 20 min, in case of a cephalea episode.

**Figure 4:**
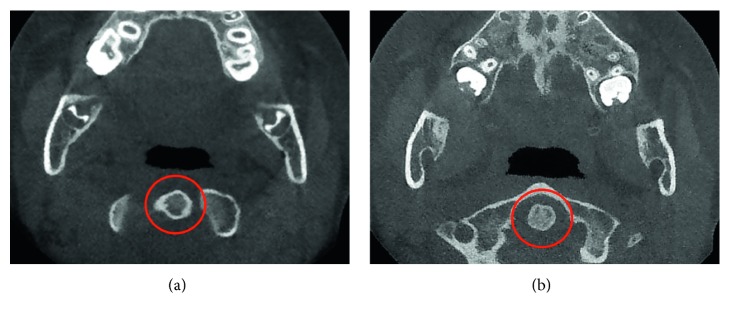
Transversal slice CT taken in closed mouth (a) and in maximum oral aperture (b). The circles represent the second cervical vertebrae's body, in the centered position.

**Figure 5:**
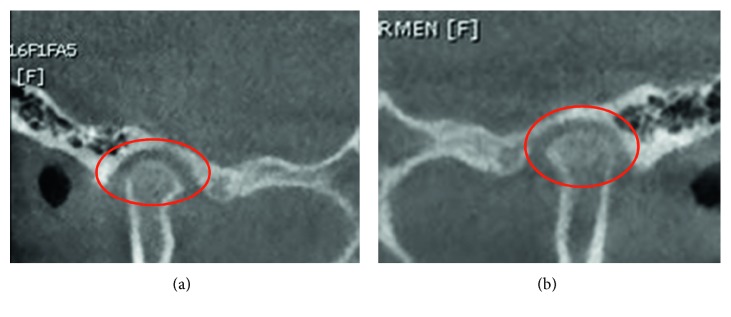
Current images of (a) left and (b) right condyles. Observe the adequate articular spaces.

The patient was scheduled regularly every two weeks. After ten months of follow-up, the parents and patient reported a significant reduction of cephalea attacks—around once per month, usually associated with scholar stress. There had been also lack of pain during chewing or on the cervical area, and no other joint dislocation episode after a maximum mouth opening was mentioned. On auscultation, it was noted that articular noises decreased notably. Paranormal oral habits were also reduced. The maximum interincisal opening is currently between 30 and 35 mm, approximately (Figure 1(b)). New CT was taken, and it showed an evident improvement (Figures 4 and 5). The girl continues under psychological management.

## 3. Discussion

Temporomandibular joint is considered as a complex synovial articulation and the most used joint in the human body. Therefore, pediatric dentistry should know the complex physiology and biomechanics of the TMJ in children and adolescents; additionally, they must always be aware of the early signs and symptoms of TMJ disorders in their patients with the aim of providing an opportune resolution and prevent progression [4].

Regarding the first issue, the TMJ is a ginglymoarthrodial joint comprising the mandibular condyle that articulates with the glenoid fossa of the temporal bone [4]. TMJ articular surfaces are in contact with the articular disc, a flexible and biconcave structure composed of a fibrous structure and nourished by synovial fluid. The articular disc is normally positioned between the posterior slope of the articular tubercle and the anterosuperior surface of the mandibular condyle. Thus, under normal conditions, the TMJ is transversely divided into two completely separated compartments [11]. On the other hand, Howard outstandingly summarizes the sequence of the different physiological processes occurring into the TMJ, during the mouth opening in children [9]. This author manifests that when the mouth is opened, the initial movement is primarily the condylar head rotating against the inferior surface of the stationary disc. Then, the disc rotates posteriorly on the condyle, and the disc-condyle system translates forward and downward, guided by contact with the disc's upper surface against the sloped articular tubercle. When the mouth is wide open, the condyle and disc gently translate together to the edge or beyond the apex of the articular tubercle.

In the second place, an exhaustive and focused clinical examination is imperative in those pediatric cases with potential TMD disorders. This examination process in children with suspected TMD, and particularly ADDR, should involve the careful assessment of three main anatomical joint structures: (a) the position of articular disc relative to the mandibular condyle; (b) the location of the condyle relative to the temporal joint surfaces; and (c) the depth of the glenoid fossa. It is also important to collect information about previous direct or indirect trauma (sometimes also called “macrotrauma”) in the orofacial area, particularly chin or TMJ injuries [19, 20]. Additionally, it is imperative an exhaustive clinical assessment: mandibular range of motion evaluation, temporomandibular joint palpation including ligaments and capsule structures, masticatory musculature (temporalis and masseter) pain under pressure, load testing, sound detection (clicking, crepitus, and hard-tissue grating); this process should be complemented with diverse auxiliary imaging (e.g., panoramic X-ray, panoramic TMJ images, cone beam computed tomography, and magnetic resonance imaging) [9].

Specifically, ADDR occurs in the closed mouth position. In this position, the articular disc is dislocated anteriorly to the condyle. However, during opening, the disc/condyle relation improves: the disc reduces by slipping back on top of the condyle [5, 10]. The local pain attributed to ADDR is related to a ligament sprain, or to muscle dysfunction, which may limit the mouth opening [5, 11].  Table 1 [11, 21, 22] lists the main different signs and symptoms that make suspicious the presence of ADDR. As in the present case, ADDR are diagnosed by clinical examination combined with diagnostic imaging [1]. According to the collected clinical information, the patient reported here belonged to the category of anterior disc displacement with reduction. The related cephalea attacks, similar to those of tension type headaches, might be caused by stimulating structures that are innervated by nerval roots from C1 to C3 [23, 24].

General and oral treatment of ADDR is still controversial. There is published evidence that has reported a self-resolution tendency even without any clinical treatment or conduct, particularly in cases with absence of pain or severe articular dysfunction [11]. When pain is present, some type of treatment is necessary, though it has not been definitely established. Different therapeutic procedures are classified as noninvasive or invasive. These treatments are mentioned in Table 2. Conservative treatments are always the first choice. Regarding the pharmaceutical management, to date, there are no approved medications for treating pain caused from TMD in children; however, NSAIDs (e.g., ibuprofen and naproxen) and muscle relaxants can be prescribed, in conjunction with oral corrective/restorative procedures, because of their relatively clinical safety [4, 7]. Additionally, the AAPD recommends the referral to an oral specialist when the following conditions are suspected: primary headaches, otitis media, allergies, abnormal posture, airway congestion, rheumatoid arthritis, connective tissue disease psychiatric disorders, or other medical anomalies [1].

In the patient reported here, the temporomandibular disorder was approached through the use of a removable occlusal splint (or bite guard). In addition, to be indicated for preventing injuries while participating in organized sports and other recreational activities, this appliance may be effective in reducing TMD symptoms and giving more comfort to the patient [1, 4, 7]. The occlusal splint is economical, lightweight, and easy to use; besides, treatment provided with these devices is noninvasive and reversible [25]. The clinical aim of the occlusal splint is to provide orthopedic stability and balance to the affected TMJ, through the modification of the relationship between the mandible and maxilla, raising the vertical dimension, and decreasing the muscle parafunctional activity (e.g. bruxism or jaw clenching) [1, 7, 18, 26]. Occlusal stabilization is achieved because all teeth are in full contact when the mouth is closed; this allows the lateral pterygoid to relax and the elevator muscles to contract, seating the mandibular condyles in centric position [1, 26]. Thus, the pressures over the TMJ are significantly reduced. Also, the device protects teeth from attrition and wear [7, 18, 25].

A possible limitation of the present case report was the lack of an initial MRI for the diagnosis of the anterior disc displacement. This tool is considered the best method to make a diagnostic assessment of the TMJ status [27]; however, MRI cost is high. Thus, in view of evident clinical findings exhibited by the patient, we decided to obtain a CT—a lower cost alternative—after a careful clinical debate between the treating specialists.

The normal function of the temporomandibular joint in children and adolescents is of fundamental importance for a normal development of the oral cavity and craniofacial region. Although the greatest TMJ structural growth takes place during the first 20 years of life, the most rapid growth occurs over the first 10 years [4].

## 4. Conclusions

Pediatric dentists are obliged to early recognize those characteristic signs and symptoms, proper to the diverse temporomandibular disorders, particularly the anterior disc displacement with reduction. An adequate diagnosis will allow to identify and apply the appropriate treatment option during the primary and mixed dentition stages, in order to avoid the progression of this condition. Among the different treatment options, the use of soft material-based occlusal splints can be recommended. This device reduces the muscular activity, thus giving more comfort to the child; moreover, the therapy relieves symptoms, changes the distribution of traumatic forces, and establish a neuromuscular harmony in the masticatory system.

## Figures and Tables

**Table 1:** Five ADDR clinical criteria validated by the research diagnostic criteria for temporomandibular disorder [11, 21, 22].

(i) Clicking during mouth opening and closure.
(ii) Interincisal distance when the clicking occurs during opening is at least 5 mm wider than interincisal distance when the clicking occurs during closure.
(iii) Clicking suppression during the mouth opening and closure (with protruded mandible).
(iv) When clicking occurs only during opening or closure, associated with clicking during mandible lateralization or protrusion.

**Table 2:** Different treatment modalities for ADDR in children and adolescents [11].

Noninvasive procedures	Invasive procedures
(i) Cognitive/behavioral therapy	(i) TMJ arthroscopies
(ii) Hot and cold therapy	(ii) Arthrocentesis
(iii) Passive and counter/resistance exercises	(iii) Surgical techniques
(iv) Relaxation techniques	
(v) Repositioning/stabilizing splints	
(vi) Biofeedback	
(vii) Ultrasound	
(viii) Phonophoresis	
(ix) Iontophoresis	
(x) Transcutaneous electrical neural	
stimulation	
(xi) Drug therapy	
(xii) Tooth selective grinding	
(xiii) Intraoral devices (removable/fix orthodontic appliances and splints)	
